# Supplementary material for: Structural and Functional Alterations in Mitochondria-Associated Membranes (MAMs) and in Mitochondria Activate Stress Response Mechanisms in an In Vitro Model of Alzheimer’s Disease
Source: Biomedicines. 2021 Jul 24;9(8):881. doi: 10.3390/biomedicines9080881 (PMC8389659; doi:10.3390/biomedicines9080881)
Supplement: Supplementary file 1 [file biomedicines-09-00881-s001.zip › biomedicines-1307823-supplementary.pdf]

Supplementary Figure

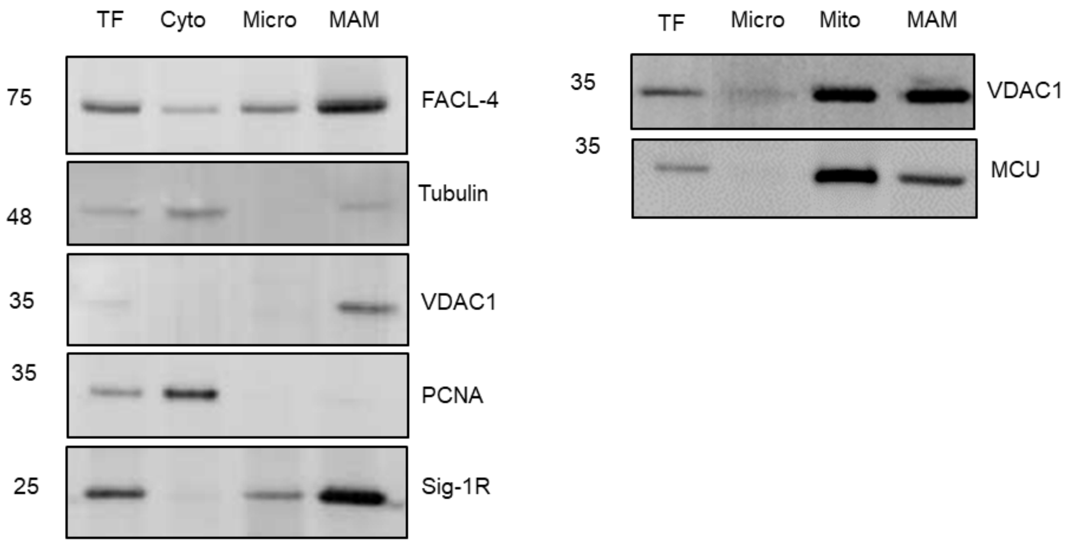

Figure S1. Analysis of protein components of subcellular fractions from wild-type (WT) mouse neuroblastoma cell line (N2A). Representative western blots for long-chain fatty-acid CoA synthase (FACL-4), tubulin, voltage-dependent anion channel 1 (VDAC1), proliferating cell nuclear antigen (PCNA), Sigma 1 receptor (Sig-1R), and mitochondrial calcium uniporter (MCU) proteins in total fraction (TF), cytosol (Cyto), microsomes (Micro), mitochondria (Mito), and mitochondrial-associated membranes (MAMs).
